# The impact of periodontal disease and dental cleaning procedures on serum and urine kidney biomarkers in dogs and cats

**DOI:** 10.1371/journal.pone.0255310

**Published:** 2021-07-29

**Authors:** Jean A. Hall, Franci J. Forman, Gerd Bobe, Giosi Farace, Murthy Yerramilli

**Affiliations:** 1 Department of Biomedical Sciences, Carlson College of Veterinary Medicine, Oregon State University, Corvallis, OR, United States of America; 2 Department of Animal and Rangeland Sciences, College of Agricultural Sciences, Oregon State University, Corvallis, OR, United States of America; 3 Linus Pauling Institute, Oregon State University, Corvallis, OR, United States of America; 4 IDEXX Laboratories, Inc., One IDEXX Drive, Westbrook, ME, United States of America; Faculty of Veterinary Medicine - University of Lisbon, PORTUGAL

## Abstract

The objective of this study was to evaluate the benefits and inherent risks of dental cleaning procedures, based on serum and urine biomarkers for kidney function and tissue damage, in dogs and cats. Thirty-one asymptomatic, mostly older dogs (14 neutered male and 17 ovariohysterectomized female dogs of various breeds between 3 and 14 years old) and cats (19 neutered male and 12 ovariohysterectomized female domestic short hair cats between 2 and 16 years old) diagnosed with periodontal disease on physical exam, and recommended by their veterinarian to have dental cleaning under general anesthesia were evaluated in a prospective study. Serum and urine samples were collected from dogs and cats 1 week before, 6 hours after, and again 1 week after the dental cleaning procedure. Samples were analyzed for biomarkers of kidney function [serum creatinine (Cr), symmetric dimethylarginine (SDMA), and blood urea nitrogen (BUN), and urine for specific gravity (USG) and protein:creatinine (UPC) ratio]. A panel of biomarkers for renal tissue damage was also assessed [serum β-aminoisobutyric acid (BAIB), and urine cystatin B and clusterin]. Samples collected one week before dental cleaning procedures showed that increased age and severity of dental disease were linked to abnormal kidney function biomarker values (age: elevated SDMA and Cr concentrations and isosthenuric USG values; disease severity: elevated UPC ratios) as well as elevated urine cystatin B and clusterin concentrations. Directly after the dental cleaning procedure, an increased number of cats with elevated SDMA concentrations was observed (specifically in cats with longer duration of dental procedures). Extended duration of dental procedures (≥60 min) was linked to increased urine cystatin B and clusterin concentrations, whereas shorter duration procedures was linked to decreased urine cystatin B and clusterin. Higher SDMA concentrations persisted in cats one week after the dental cleaning procedures and were linked to elevated UPC ratios one week before cleaning procedures. In conclusion, the results of this study indicate a link between severity of dental disease, renal tissue injury, and impaired renal function. Longer duration dental procedures in cats may carry inherent risks of kidney injury and impaired renal function.

## Introduction

Chronic kidney disease (CKD) is a progressive disease that affects middle-aged and older cats and dogs. Prevalence estimates of CKD vary between 0.05% and 3.74%, including a prevalence of 0.4% and 1.2% among dogs and cats, respectively, attending UK primary care practices [1, 2]. Disease prevalence is likely underestimated, as CKD is a clinically silent disease in its early, most treatable stages. Moreover, CKD diagnosis is based on consistent changes in multiple serum and urine biomarkers [creatinine (Cr), symmetric dimethylarginine (SDMA), blood urea nitrogen (BUN), urine specific gravity (USG), urine protein:creatinine (UPC) ratio] over multiple, routine veterinary visits [3]. Data from IDEXX Laboratories in the USA, from more than 3 million dogs and more than 1 million cats measured after July 2015 reported elevated SDMA concentrations, indicative of CKD, in approximately 7 to 10% of dogs 8 to 10 years of age [4], in over 40% for cats 5 to 15 years of age [5], and in 80% of cats over 15 years of age [5].

Older dogs and cats require regular veterinary visits for diagnosis and management of periodontal disease. One study diagnosed periodontitis in 82% of dogs aged 6 to 8 years of age and 96% of dogs aged 12 to 14 years [6]. Small-breed dogs are more susceptible to periodontal disease, with more than 85% reportedly having periodontal disease [7]. In cats, disease prevalence is likely underestimated as general anesthesia is required for an accurate diagnosis [8]. One study diagnosed periodontal disease in 50% of cats over 4 years of age, and 93% of cats over 8 years of age showed radiographic signs of periodontal bone or root loss [9].

Because CKD is often a clinically silent disease in its early stages, routine veterinary visits for periodontal disease provide an opportunity to assess renal function and detect early stages of CKD in cats and dogs that appear physically healthy. Periodontal disease results in inflammation of the deep supporting structures of the teeth. Chronic inflammation of the gums and oral cavity is associated with transient bacteremia following chewing, tooth brushing, and dental or surgical interventions [10]. This can lead to pathologic changes in distant organs, including the kidneys [7]. A 1.4 × greater likelihood of kidney pathology has been reported for each square centimeter of periodontal disease burden in dogs [11]. Periodontal disease may result in deposition of immune complexes in the kidneys of dogs and cats leading to glomerulonephritis [7]. Pyelonephritis or interstitial nephritis may also arise secondary to the associated bacteremia [7].

Additionally, dental cleaning procedures performed under general anesthesia carry an inherent risk of kidney injury even in healthy patients, from hypotension and nephrotoxic drugs, despite supportive care including intravenous fluids and regular monitoring of vital signs [12]. Recently developed kidney-specific inflammation biomarkers, including serum β-aminoisobutyric acid (BAIB), and urine cystatin B and kidney-specific clusterin, may indicate kidney inflammation and tissue damage associated with periodontal disease, dental cleaning procedures under general anesthesia, or both [7].

The objective of the current study was to investigate benefits and inherent risks of dental cleaning procedures on kidney health in dogs and cats using serum and urine biomarkers for kidney function and tissue damage. Our three hypotheses were: 1) Dogs and cats recommended for dental cleaning procedures [i.e., dental scaling and polishing to remove plaque and tartar deposits from all tooth surfaces with or without tooth extraction(s)] and otherwise normal physical exams may have impaired kidney function, kidney inflammation and tissue damage, or both one week before the procedure. 2) Dental cleaning procedures performed under general anesthesia for extended periods of time (i.e., ≥ 60 min vs. < 60 min) may impact biomarkers of renal function and acute inflammation and tissue damage measured six hours after the procedure. 3) Dental cleaning procedures performed under general anesthesia for extended periods of time (i.e., ≥ 60 min vs. < 60 min) may continue to impact biomarkers of renal function and chronic inflammation and tissue damage measured one week after the procedure.

## Methods

### Animal ethics statement and study design

This was a prospective study utilizing a “before–and–after” study design. There was one participating clinic in Corvallis, OR with five participating veterinarians in that clinic. To be eligible for enrollment, dogs and cats had to appear healthy based on their physical exam with no evidence of underlying disease (other than periodontal disease), and be recommended by their veterinarian to have dental cleaning procedures under general anesthesia. Owners signed an informed consent form before enrollment of their dog or cat and had to agree to comply with the instructions given by their veterinarian and listed in the consent form. Owners were compensated for their participation in the study by reduced laboratory fees. The study protocol was reviewed by the Oregon State University Animal Care and Use Committee and considered exempt from IACUC review as the collection time points of samples used in this study were part of the clinical standard of care. All pets remained with their owners throughout the study.

A total of 35 dogs were enrolled between November 2016 and October 2017, and 34 cats were enrolled between January 2017 and June 2018. Blood and urine samples were collected (after withholding food overnight) one week prior to general anesthesia and dental cleaning procedures, six hours post dental procedures, and again one week post dental procedures. Four dogs and 3 cats were removed from the study, because they did not complete all three blood and urine sample collection times.

A total of 31 dogs (14 neutered males and 17 ovario-hysterectomized females) of various breeds and 31 domestic short hair cats (19 neutered males and 12 ovario-hysterectomized females) were included in the analysis: Most cats and dogs were middle-aged or older. All but one 3-year-old female dog were between 6.5 and 14-years-old, and all but one 2-year-old male cat and one 3-year-old male cat were between 5 and 16-years-old. Most dogs were medium-sized (10 to 30 kg; 56%); the rest were under 10 kg (37%) or above 30 kg (7%). Cats weighed between 2.4 and 9.8 kg (mean: 5.2 kg).

All animals were administered general anesthesia for the dental procedure. The type of pre-anesthetic drugs used and the type of general anesthesia administered (all pets were induced with propofol and maintained with sevoflurane) were recorded as was the length of time under general anesthesia. The median anesthesia time (range in parenthesis) in dogs was 60 min (25 to 205 min) and in cats was 60 min (15 to 180 min). Twenty-one dogs and 16 cats had general anesthesia lasting at least 60 minutes. The type of procedure performed was also recorded (e.g., dental scaling and polishing to remove the plaque and tartar from all tooth surfaces ± the number of teeth extracted). Sixteen dogs and 16 cats had at least one tooth extracted. In addition, the volume and rate of intravenous fluids administered, and the use of post-operative analgesics and antibiotics (dogs: cefpodoxime, n = 2; metronidazole, n = 1; cats: clindamycin, n = 2; orbifloxacin, n = 1; amoxicillin, n = 1) were recorded. All dogs with extractions received carprofen and cats with extractions received buprenorphine at clinically recommended analgesic dosages. The median intravenous fluids administration rate (range in parenthesis) in dogs were 1.5 mL/kg/h (0.4 to 5.2 mL/kg/h) and in cats were 2.1 mL/kg/h (0. 9 to 4.9 mL/kg/h). Four dogs and six cats received intravenous fluids administered at a rate of 3 to 6 mL/kg/h, but none greater than 6 mL/kg/h [13].

### Serum and urine analyses

Serum was separated from blood after centrifugation at 1300 *g* for 10 min at room temperature. Serum and urine samples (urine collected by cystocentesis) were split into two aliquots. One aliquot of serum and urine was shipped immediately to an IDEXX reference laboratory (Sacramento, CA) to assess biomarkers of kidney function. To exclude non-renal conditions, a complete blood count, serum chemistry panel, thyroid panel, urinalysis, USG, UPC ratio, and urine culture were analyzed at each collection point. Biomarkers of kidney function included serum Cr, BUN and SDMA concentrations, and urine for USG and UPC ratio. Standard serum BUN concentrations were determined using enzymatic colorimetric methods, Cr concentrations were measured using the Jaffe method, and serum SDMA concentrations were measured using a validated, commercially available, high-throughput immunoassay (IDEXX SDMA^®^ Test, IDEXX Laboratories, Inc.). Urine specific gravity was determined using a refractometer. Urine concentrations of protein (benzethonium chloride turbidimetric method) and Cr (same assay as serum Cr) were measured and the UPC ratios are reported as mg/dL protein: mg/dL creatinine.

The other aliquots of serum and urine were shipped to the IDEXX research laboratory (Westbrook, ME) where they were stored at 80ᵒC for subsequent batched analyses. Serum BAIB was quantified using liquid chromatography mass spectrometry (LCMS) [7]. Concentrations of urine cystatin B and kidney-specific clusterin were measured using validated, patented, high-throughput sandwich format immunoassays [7].

### Chronic kidney disease staging and reference ranges for serum and urine kidney biomarkers

Dogs and cats with CKD were staged according to guidelines developed by the International Renal Interest Society (IRIS) [14] and accepted by the American and European Societies of Veterinary Nephrology and Urology [3]. Pets were considered reasonable to have CKD if one or more of these diagnostic findings were observed: 1) persistently increased Cr (Cr ≥ 1.4 mg/dL in dogs and Cr ≥ 1.6 mg/dL in cats), 2) persistently increased SDMA > 14 μg/dL, or 3) persistently dilute urine (USG ≤ 1.020 in dogs and USG ≤ 1.030 in cats) without identifiable non-renal cause. None of the pets had kidney imaging performed. Proteinuria was classified as follows: UPC ratio 0.2 to 0.5 = borderline proteinuric for dogs; UPC ratio 0.2 to 0.4 = borderline proteinuric for cats; UPC ratio >0.5 = proteinuric for dogs; UPC ratio >0.4 = proteinuric for cats. Blood pressure measurement were not consistently performed, and therefore, are not reported.

In this study, dogs with normal Cr concentrations (<1.4 mg/dL) and elevated SDMA concentrations (> 14 μg/dL), or with persistently dilute urine (USG ≤ 1.020) without identifiable non-renal cause, confirmed at multiple consecutive assessments were classified as IRIS-Stage 1 CKD. If serum SDMA was persistently >18μg/dL in a dog whose Cr was <1.4 mg/dL, this canine patient was staged as IRIS CKD Stage 2. Dogs with serum Cr concentrations between 1.4–2.8 mg/dL and with SDMA concentrations between 18–35 μg/dL were classified as IRIS-Stage 2 CKD.

In this study, cats with serum Cr concentrations (<1.6 mg/dL) and elevated SDMA concentrations (> 14 μg/dL), or with persistently dilute urine (USG ≤ 1.030) without identifiable non-renal cause, confirmed at multiple consecutive assessments were classified as IRIS-Stage 1 CKD. If serum or plasma SDMA was persistently >18 μg/dL in a cat whose Cr was <1.6 mg/dL, this feline patient was staged as IRIS CKD Stage 2. Cats with serum Cr concentrations between 1.6–2.8 mg/dL and with SDMA concentrations between 18–25 μg/dL were classified as IRIS-Stage 2 CKD. Cats with serum Cr concentrations between 2.9–5.0 mg/dL and with SDMA concentrations between 26–38 μg/dL were classified as IRIS-Stage 3 CKD.

### Statistical analysis

Data were statistically analyzed using SAS version 9.4 (version 5.0, SAS Institute Inc., Cary, NC). Most of the data were not normally distributed. Thus, we used non-parametric tests. We examined kidney biomarker concentrations and changes in kidney biomarker concentrations from one week before the dental procedure using the Wilcoxon rank sum test and the paired Wilcoxon rank sum test, respectively. In addition, we counted the number of animals with biomarker concentrations outside the reference ranges established by IDEXX and compared them using the Fisher’s Exact test. We compared cats and dogs. In addition, we compared overall and separately within cats and dogs, females with males, older vs. younger animals (≥ 9 years old vs. < 9 years old), animals with vs. without extended anesthesia (≥ 60 min vs < 60 min), animals with vs. without tooth extraction(s), and animals with vs. without abnormal kidney function biomarkers. Spearman rank correlation coefficients were used to examine the link between anesthesia length of time (in min) or age (in years) with kidney biomarker concentrations. All statistical tests were two-sided. Significance was declared at *P* ≤ 0.05 and a statistical tendency was declared at 0.05 < *P* < 0.10.

## Results

### Kidney biomarker concentrations in dogs and cats one week before dental procedures

#### Kidney function biomarkers

**Fig 1:**
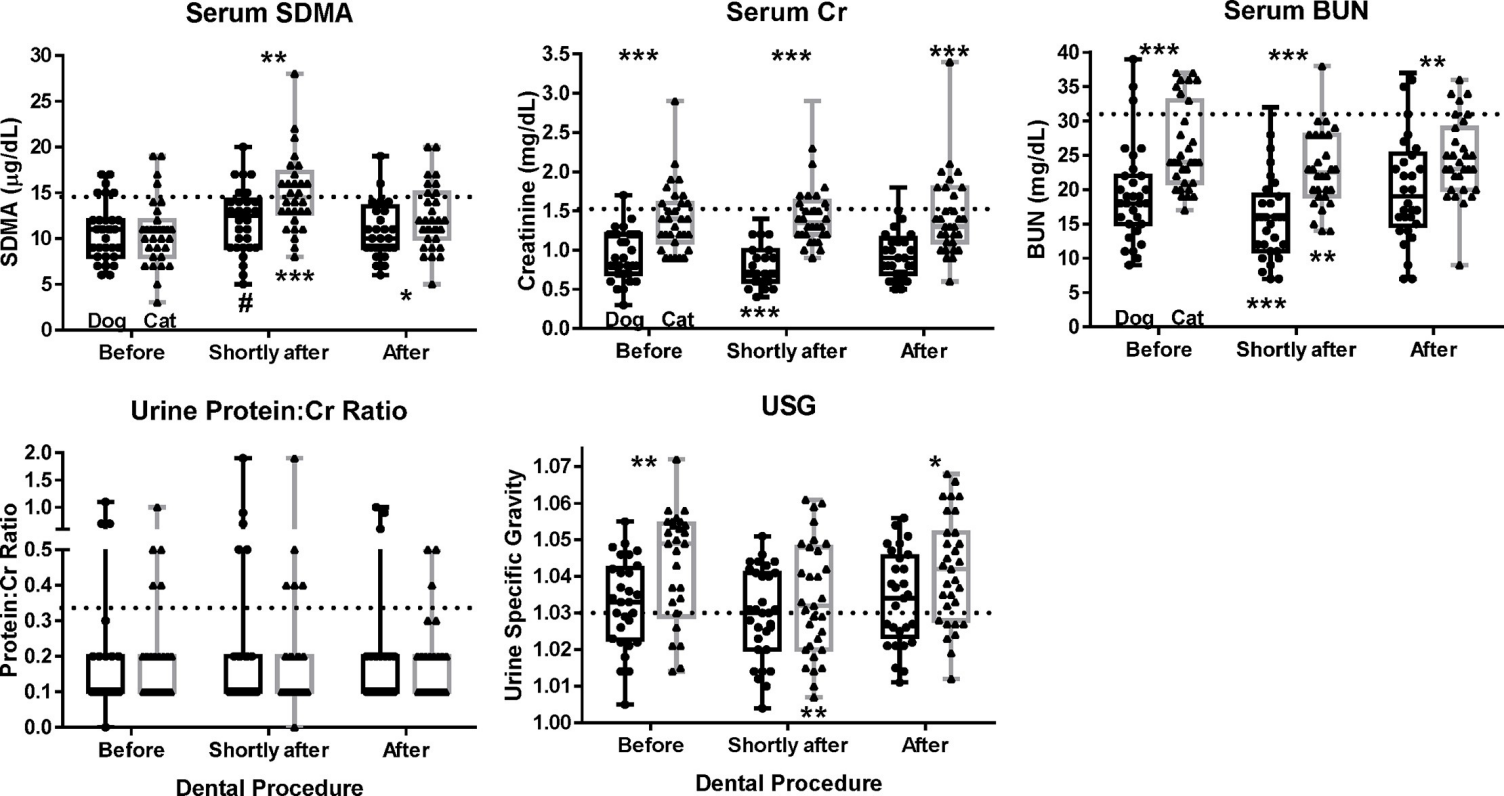
Serum and urine kidney function biomarker values before and after general anesthesia and dental cleaning procedures in cats and dogs with periodontal disease (n = 62). Serum and urine samples were collected from dogs and cats 1 week before, 6 hours after, and again 1 week after dental cleaning procedures. Samples were analyzed for serum creatinine (Cr), serum symmetric dimethylarginine (SDMA), and blood urea nitrogen (BUN), and urine for specific gravity (USG) and protein:creatinine (UPC) ratio. Results are shown as box and whiskers plots with individual points shown as filled circles for dogs and filled triangles for cats. The horizontal lines from bottom to top indicate minimum, 25^th^ percentile, median, 75^th^ percentile and maximum. Above the box and whiskers plots, differences between cats and dogs are denoted with a # for 0.10 ≤ *P* ≤ 0.05, a * for 0.05 ≤ *P* ≤ 0.01, a ** for 0.01 ≤ *P* ≤ 0.001, and a *** for *P* ≤ 0.001 and were calculated using the Wilcoxon rank sum test. Below the box and whiskers plots, changes from 1 week before dental procedures are denoted with a # for 0.10 ≤ *P* ≤ 0.05, a * for 0.05 ≤ *P* ≤ 0.01, a ** for 0.01 ≤ *P* ≤ 0.001, and a *** for *P* ≤ 0.001 and were calculated using a paired Wilcoxon rank sum test.

We examined five kidney function biomarkers (**Fig 1; Table 1**): serum SDMA, Cr, and BUN as well as urine UPC ratio and USG. Serum SDMA concentrations ranged from 6 to 17 μg/dL in dogs and from 3 to 19 μg/dL in cats. The normal reference interval for SDMA used by IDEXX is 0 to 14 μg/dL in both species. Six dogs (7, 9, 9, 11, and 14-year-old males and a 10-year-old female) and four cats (9 and 12-year-old males and 14 and 15-year-old females) had SDMA concentrations outside the normal reference interval.

**Table 1:** Serum and urine kidney function biomarkers before and after general anesthesia and dental cleaning procedures in cats and dogs with periodontal disease (n = 62)*.

Biomarkers of	Dental cleaning procedures			*P*-values^‡^	
Kidney Function	1 week before	6 hours after	1 week after	6 hours after	1 week after
Dogs					
Serum					
Creatinine, mg/dL	0.8 (0.7, 1.2)***	0.7 (0.6, 1.0)***	0.9 (0.7, 1.1)***	<0.0001***	0.39
SDMA^§^, μg/dL	11 (8, 12)	12.5 (9, 14)*	10 (9, 13)	0.08**	0.97
BUN^§^, mg/dL	18 (15, 22)***	16 (11, 19)***	19 (15, 25)*	<0.0001	0.95
Urine					
UPC^§^ (ratio)	0.1 (0.1, 0.2)	0.1 (0.1, 0.2)	0.1 (0.1, 0.2)	0.14*	0.96
USG^§^	1.033 (1.023, 1.042)**	1.030 (1.020, 1.041)	1.034 (1.025, 1.045)*	0.44^†^	0.71
Cats					
Serum					
Creatinine, mg/dL	1.4 (1.1, 1.6)	1.35 (1.2, 1.6)	1.3 (1.1, 1.8)	0.15	0.46
SDMA^§^, μg/dL	11 (8, 12)	14.5 (13, 17)	12 (10, 15)	<0.0001	0.02
BUN^§^, mg/dL	24 (21, 33)	22.5 (19, 28)	23 (20, 29)	0.007	0.12
Urine					
UPC^§^ (ratio)	0.1 (0.1, 0.2)	0.1 (0.1, 0.2)	0.1 (0.1, 0.2)	0.31	0.34
USG^§^	1.049 (1.030, 1.054)	1.032 (1.020, 1.048)	1.042 (1.028, 1.052)	0.002	0.71

*Median (Q25, Q75). 14 male neutered dogs, 17 female spayed dogs; 9.5 ± 2.1 years (range: 3–14 years old. 19 male neutered cats, 12 female spayed cats; 9.7 ± 3.5 years (range: 2–16 years old).

^‡^A paired Wilcoxon rank sum test was used to determine *P* values for changes from 1 week before dental procedures.

Dog and cat species differences were calculated using Wilcoxon rank sum test, with 0.05 < *P* < 0.10 denoted by ^†^, 0.01 < *P* < 0.05 denoted by *, 0.001 < *P* < 0.01 denoted by **, and *P* < 0.001 denoted by ***.

^§^SDMA, symmetric dimethylarginine; BUN, blood urea nitrogen; UPC, urine protein creatinine ratio; USG, urine specific gravity.

Serum Cr concentrations ranged from 0.3 to 1.7 mg/dL in dogs and from 0.9 to 2.9 mg/dL in cats. The normal reference intervals for Cr used by IDEXX are 0.5 to 1.5 mg/dL in dogs and 0.9 to 2.3 mg/dL in cats. Two dogs (9-year-old male with 0.3 mg/dL and 11-year-old male with 1.7 mg/dL) and one cat (9-year-old male with 2.9 mg/dL) had Cr concentrations outside the normal reference intervals. In addition, nine cats (8, 8, 9, 10, 11, 12, and 13-year-old males and 14 and 15 year-old-females) had Cr concentrations >1.6 mg/dL), a cut-off consistent with IRIS guidelines [3].

Serum BUN concentrations ranged from 9 to 39 mg/dL in dogs and from 19 to 37 mg/dL in cats. The normal reference intervals for BUN used by IDEXX are 9 to 31 mg/dL in dogs and 16 to 27 mg/dL in cats. Three dogs had BUN concentrations outside the normal reference interval (11, 12, and 14-year-old males with 39, 33, and 35 mg/dL, respectively). In addition, eight cats (5, 7, 8, 9, 11, and 13-year-old males and 15 and 16 year-old-females) had elevated BUN concentrations (33–37 mg/dL) that clustered separately from the other cats.

Urine UPC ratios ranged from 0.0 to 1.1 in dogs and from 0.1 to 1.0 in cats. Three dogs (UPC > 0.5; 8 and 12-year-old males and 7-year-old female) and three cats (UPC > 0.4; 5 and 7-year-old males and 16-year-old female) had proteinuria. Six dogs (UPC: 0.2–0.5; one 14-year-old male and 9, 9, 10, 12, and 12 year-old females) and ten cats (UPC: 0.2–0.4; 7, 9, and 13-year-old males and 6, 7, 12, 13, 14, 14, 15-year-old females) had borderline proteinuria [3].

Urine USG ranged from 1.005 to 1.055 in dogs and from 1.014 to 1.072 in cats. Four dogs (USG ≤ 1.020; 3, 7, 8, and 10-year-old females) and nine cats (USG ≤ 1.030; 8, 9, and 12-year-old males and 7, 10, 12, 14, 15, 16-year-old females) were isosthenuric [3].

A total of 18 dogs and 16 cats had at least one abnormal kidney function biomarker. Serum SDMA and Cr concentrations were correlated (overall: r = +0.34; *P* = 0.008; dogs: r = +0.36; *P* = 0.05; cats: r = +0.47; *P* = 0.008) and BUN and UPC concentrations were correlated (overall: r = +0.35; *P* = 0.005; dogs: r = +0.33; *P* = 0.07; cats: r = +0.45; *P* = 0.01). Three dogs and five cats had at least two abnormal kidney function biomarkers. There were two groups of animals with abnormal kidney function biomarkers: those animals with high SDMA concentrations and those with proteinuria. All four cats with high SDMA concentrations also had isosthenuria, and two of the six dogs with high SDMA concentration had another abnormal kidney function biomarker (two had increased BUN and one had increased Cr); however none of the 10 animals with high SDMA concentrations had proteinuria. Of the six animals with proteinuria, one dog and one cat also had isosthenuria but none had high Cr, SDMA, or BUN concentrations.

#### Species and sex differences

Serum Cr and BUN concentrations and USG values were higher in cats than in dogs (**Fig 1**). Isosthenuria was more common in females than males (10 vs. 3; *P* = 0.03) as was borderline proteinuria (12 vs. 4; *P* = 0.01; **Table 2**). In contrast, proteinuria and abnormal Cr, SDMA, or BUN concentrations were more common in male than female dogs (8 vs. 2; *P* = 0.02); similar trends were observed in male vs. female cats when cats with elevated Cr or BUN concentrations were included (7 vs. 3).

**Table 2:** Serum and urine kidney function biomarkers before and after general anesthesia and dental cleaning procedures in cats and dogs with periodontal disease stratified by sex (n = 62)*.

	Dental cleaning procedures			*P*-values^‡^	
	1 week before	6 hours after	1 week after	6 hours after	1 week after
Serum					
Creatinine, mg/dL					
Female	1.0 (0.8, 1.3)^†^	0.95 (0.65, 1.3)*	1.1 (0.8, 1.4)	0.04	0.27
Male	1.2 (0.9, 1.6)	1.2 (0.9, 1.5)	1.15 (0.9, 1.45)	0.66	0.72
SDMA, μg/dL					
Female	11 (9, 12)	13 (10, 15.5)	11 (9, 13.5)	0.0006	0.48
Male	11 (8, 12)	13.5 (11, 17)	11 (9, 14.5)	0.0001	0.17
BUN, mg/dL					
Female	21 (18, 25)	17 (12, 22.5)	23 (17, 25)	<0.0001	0.87
Male	22 (19, 33)	20 (16, 25.5)	22 (19.5, 30.5)	0.002	0.34
Urine^§^					
UPC (ratio)					
Female	0.1 (0.1, 0.2)	0.1 (0.1, 0.2)	0.1 (0.1, 0.2)	0.39	0.39
Male	0.1 (0.1, 0.1)	0.1 (0.1, 0.2)	0.1 (0.1, 0.2)	0.90	0.43
USG					
Female	1.030 (1.021, 1.043)*	1.026 (1.015, 1.041)^†^	1.036 (1.023, 1.046)	0.25	0.51
Male	1.045 (1.032, 1.053)	1.033 (1.027, 1.044)	1.038 (1.028, 1.049)	0.01	0.45

*Median (Q25, Q75). 14 male neutered dogs, 17 female spayed dogs; 9.5 ± 2.1 years (range: 3–14 years old. 19 male neutered cats, 12 female spayed cats; 9.7 ± 3.5 years (range: 2–16 years old).

^‡^A paired Wilcoxon rank sum test was used to determine *P* values for changes from 1 week before the procedure.

Male and female differences were calculated using Wilcoxon rank sum test, with 0.05 < *P* < 0.10 denoted by ^†^, 0.01 < *P* < 0.05 denoted by *, 0.001 < *P* < 0.01 denoted by **, and *P* < 0.001 denoted by ***.

^§^SDMA, symmetric dimethylarginine; BUN, blood urea nitrogen; UPC, urine protein creatinine ratio; USG, urine specific gravity.

#### Age effects

The number of animals with elevated SDMA, Cr, or BUN increased with age (**Table 3**). Abnormally high SDMA concentrations were only observed in cats ≥ 9-years-old (6 of 18 ≥ 9-years-old cats; *P* = 0.03), and elevated serum Cr concentrations were only observed in cats ≥ 8-years-old (10 of 20 ≥ 8-years-old cats; *P* = 0.005). More ≥ 9-years-old animals tended to have elevated SDMA, Cr, or BUN concentrations compared with younger animals (10 of 41 ≥ 9-years-old animals vs. 1 of 21 younger animals; *P* = 0.08). In contrast, USG values decreased with age in cats (spearman r = -0.55; *P* = 0.002), but not in dogs (spearman r = +0.06; *P* = 0.76).

**Table 3:** Serum and urine kidney function biomarkers before and after general anesthesia and dental cleaning procedures in cats and dogs with periodontal disease stratified by age (n = 62)*.

	Dental cleaning procedures			*P*-values^‡^	
	1 week before	6 hours after	1 week after	6 hours after	1 week after
Biomarkers of Kidney Function					
Serum					
Creatinine, mg/dL					
<9 years old	1.0 (0.9, 1.2)	1.2 (0.8, 1.35)	1.0 (0.8, 1.1)*	0.37*	0.27*
≥9 years old	1.2 (0.8, 1.4)	1.0 (0.7, 1.4)	1.2 (1.0, 1.5)	0.003	0.05
SDMA, μg/dL					
<9 years old	9 (8, 11)^†^	11.5 (10, 14)*	11 (9, 11)	0.004	0.29
≥9 years old	11 (9, 14)	14 (12, 17)	12 (9, 15)	<0.0001	0.31
BUN, mg/dL					
<8 years old	20 (19, 24)	19.5 (13.5, 22.5)	20 (16, 24)*	0.01	0.05
≥8 years old	23 (18, 27)	19 (15.5, 24)	23 (19.5, 28.5)	<0.0001	0.86
Urine^§^					
UPC (ratio)					
<9 years old	0.1 (0.1, 0.2)	0.1 (0.1, 0.2)	0.1 (0.1, 0.2)	0.29^†^	0.04^†^
≥9 years old	0.1 (0.1, 0.2)	0.1 (0.1, 0.2)	0.1 (0.1, 0.2)	0.09	0.86
USG					
<9 years old	1.050 (1.021, 1.055)	1.031 (1.020, 1.041)	1.047 (1.026, 1.054)	0.03	0.66
≥9 years old	1.034 (1.028, 1.046)	1.030 (1.023, 1.043)	1.037 (1.027, 1.044)	0.08	0.65

*Median (Q25, Q75). 14 male neutered dogs, 17 female spayed dogs; 9.5 ± 2.1 years (range: 3–14 years old. 19 male neutered cats, 12 female spayed cats; 9.7 ± 3.5 years (range: 2–16 years old).

^‡^A paired Wilcoxon rank sum test was used to determine *P* values for changes from 1 week before the procedure.

Differences due to age of the animal were calculated using Wilcoxon rank sum test, with 0.05 < *P* < 0.10 denoted by ^†^, 0.01 < *P* < 0.05 denoted by *, 0.001 < *P* < 0.01 denoted by **, and *P* < 0.001 denoted by ***.

^§^SDMA, symmetric dimethylarginine; BUN, blood urea nitrogen; UPC, urine protein creatinine ratio; USG, urine specific gravity.

#### Abnormal kidney function biomarkers are linked with severity of dental disease

Disease severity one week prior to dental procedures was determined retrospectively by classifying pets according to subsequent procedures performed. Three parameters were assessed to evaluate dental disease severity: length of anesthesia (in min), extended anesthesia time (<60 min vs. ≥60 min), and type of dental procedure (cleaning with vs. without tooth extraction). Extended anesthesia time and tooth extraction(s) were linked. Only one of 16 cats with tooth extraction had anesthesia time <60 min, and only one of 15 cats without tooth extraction had anesthesia time ≥60 min. Three of 15 dogs with tooth extraction had anesthesia time <60 min and nine of 16 dogs without tooth extraction had anesthesia time ≥60 min.

Severity of dental disease one week prior to dental procedures (based on subsequent procedures performed) and UPC ratios were linked (**Table 4**). Length of subsequent anesthesia time was correlated with UPC ratios (overall: r = +0.29; *P* = 0.02; dogs: r = +0.33; *P* = 0.07; cats: r = +0.30; *P* = 0.10). Higher UPC ratios (overall: *P* = 0.01; dogs: *P* = 0.09; cats: *P* = 0.03) were observed in animals with vs. without ≥60 min anesthesia. Elevated UPC ratios were observed in eight of 21 dogs and ten of 16 cats with ≥60 min anesthesia vs. one of 10 dogs and three of 15 cats with <60 min anesthesia (*P* = 0.01). We tended to observe higher UPC ratios (overall: *P* = 0.07; dogs: *P* = 0.38; cats: *P* = 0.10) in animals with vs. without tooth extraction.

**Table 4:** Serum and urine kidney function biomarkers before and after general anesthesia and dental cleaning procedures in cats and dogs with periodontal disease stratified by anesthesia length (n = 62)*.

	Dental cleaning procedures			*P*-values^‡^	
	1 week before	6 hours after	1 week after	6 hours after	1 week after
Biomarkers of Kidney Function					
Serum					
Creatinine, mg/dL					
<60 min Anesth.	1.2 (0.9, 1.4)	1.2 (0.9, 1.3)	1.1 (1.0, 1.5)	0.75	0.29
≥60 min Anesth.	1.1 (0.8, 1.4)	1.0 (0.6, 1.4)	1.1 (0.8, 1.3)	0.04	0.73
SDMA, μg/dL					
<60 min Anesth.	11 (9, 12)	14 (11, 16)	11 (9, 12)	0.0007	0.88
≥60 min Anesth.	10 (8, 12)	13 (10, 16)	11 (9, 16)	<0.0001	0.05
BUN, mg/dL					
<60 min Anesth.	21 (18, 24)	19 (16, 22)	23 (18, 25)	0.004	0.77
≥60 min Anesth.	23 (18, 33)	20 (14, 28)	22.5 (17, 28.5)	<0.0001	0.18
Urine^§^					
UPC (ratio)					
<60 min Anesth.	0.1 (0.1, 0.1)*	0.1 (0.1, 0.1)***	0.1 (0.1, 0.1)*	0.50	0.84
≥60 min Anesth.	0.1 (0.1, 0.2)	0.2 (0.1, 0.2)	0.1 (0.1, 0.2)	0.45	0.15
USG					
<60 min Anesth.	1.042 (1.028, 1.053)	1.029 (1.020, 1.047)	1.045 (1.032, 1.056)*	0.06	0.14*
≥60 min Anesth.	1.035 (1.026, 1.047)	1.031 (1.025, 1.040)	1.035 (1.026, 1.044)	0.04	0.20

*Median (Q25, Q75). 14 male neutered dogs, 17 female spayed dogs; 9.5 ± 2.1 years (range: 3–14 years old. 19 male neutered cats, 12 female spayed cats; 9.7 ± 3.5 years (range: 2–16 years old).

^‡^A paired Wilcoxon rank sum test was used to determine *P* values for changes from 1 week before the procedure.

Differences due to anesthesia length were calculated using Wilcoxon rank sum test, with 0.05 < *P* < 0.10 denoted by ^†^, 0.01 < *P* < 0.05 denoted by *, 0.001 < *P* < 0.01 denoted by **, and *P* < 0.001 denoted by ***.

^§^Anesth., anesthesia; SDMA, symmetric dimethylarginine; BUN, blood urea nitrogen; UPC, urine protein creatinine ratio; USG, urine specific gravity.

#### Tissue damage biomarkers in dogs and cats recommended for dental procedures

**Fig 2:**
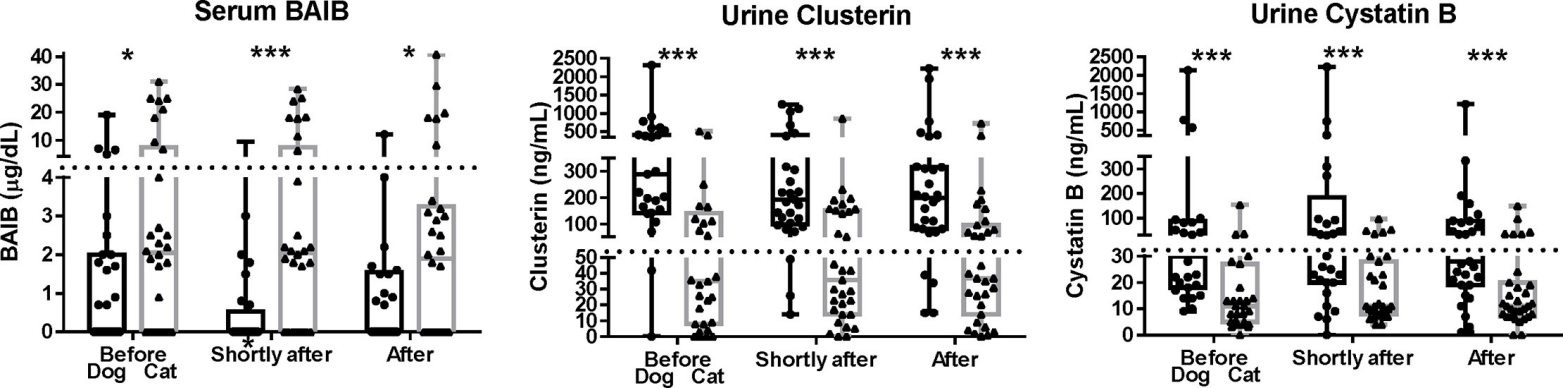
Serum and urine kidney tissue damage biomarker concentrations before and after general anesthesia and dental cleaning procedures in cats and dogs with periodontal disease (n = 62). Serum and urine samples were collected from dogs and cats 1 week before, 6 hours after, and again 1 week after dental cleaning procedures. Samples were analyzed for serum β-aminoisobutyric acid (BAIB), and urine for cystatin B and clusterin. Results are shown as box and whiskers plots with individual points shown as filled circles for dogs and filled triangles for cats. The horizontal lines from bottom to top indicate minimum, 25^th^ percentile, median, 75^th^ percentile and maximum. Above the box and whiskers plots, differences between cats and dogs are denoted with a # for 0.10 ≤ *P* ≤ 0.05, a * for 0.05 ≤ *P* ≤ 0.01, a ** for 0.01 ≤ *P* ≤ 0.001, and a *** for *P* ≤ 0.001 and were calculated using the Wilcoxon rank sum test. Below the box and whiskers plots, changes from 1 week before dental procedures are denoted with a # for 0.10 ≤ *P* ≤ 0.05, a * for 0.05 ≤ *P* ≤ 0.01, a ** for 0.01 ≤ *P* ≤ 0.001, and a *** for *P* ≤ 0.001 and were calculated using a paired Wilcoxon rank sum test.

Three potential kidney tissue damage biomarkers were measured in dogs and cats: serum BAIB and urine cystatin B and kidney-specific clusterin (**Fig 2; Table 5**). Serum BAIB concentrations ranged from 0 to 19 μg/dL in dogs and from 0 to 31 μg/dL in cats. Using serum BAIB concentrations, pets were stratified into three groups: non-detectable (16 dogs and 10 cats), low (0.1–4 μg/dL; 10 dogs and 12 cats); and high (>4 μg/dL; 4 dogs and 8 cats).

**Table 5:** Serum and urine kidney tissue damage biomarkers before and after general anesthesia and dental cleaning procedures in cats and dogs with periodontal disease (n = 62)*.

Biomarkers of	Dental cleaning procedures			*P*-values^‡^	
Kidney Damage	1 week before	6 hours after	1 week after	6 hours after	1 week after
Dogs					
Serum (μg/dL)					
BAIB^§^	0 (0, 2.0)*	0 (0, 0.35)***	0 (0, 1.5)*	0.03	0.23
Urine (ng/mL)^§^					
Cystatin B	30 (18, 90)***	31 (20, 97)**	28 (19, 89)***	0.43	0.79
Clusterin	289 (142, 419)***	193 (103, 378)***	199 (81, 315)***	0.93	0.21
Cats					
Serum (μg/dL)					
BAIB	2.05 (0, 6.8)	2.05 (0, 6.2)	1.9 (0, 3.2)	0.57	0.45
Urine (ng/mL)^§^					
Cystatin B	11 (5, 27)	11 (8, 28)	11 (7, 20)	0.19	0.92
Clusterin	35 (8, 120)	36 (14, 151)	37 (14, 95)	0.94	0.69

*Median (Q25, Q75). 14 male neutered dogs, 17 female spayed dogs; 9.5 ± 2.1 years (range: 3–14 years old. 19 male neutered cats, 12 female spayed cats; 9.7 ± 3.5 years (range: 2–16 years old).

^‡^A paired Wilcoxon rank sum test was used to determine *P* values for changes from 1 week before the procedure.

Dog and cat species differences were calculated using Wilcoxon rank sum test, with 0.05 < *P* < 0.10 denoted by ^†^, 0.01 < *P* < 0.05 denoted by *, 0.001 < *P* < 0.01 denoted by **, and *P* < 0.001 denoted by ***.

^§^BAIB, ß-aminoisobutyric acid.

Urine clusterin concentrations ranged from 0 to 2313 ng/mL in dogs and from 0 to 515 ng/mL in cats. Using urine clusterin concentrations, pets were classified as low (≤150 ng/mL for dogs and ≤50 ng/mL for cats; 7 dogs and 17 cats); elevated (151–350 ng/mL for dogs and 51–150 ng/mL for cats; 8 dogs and 5 cats), and high (>350 ng/mL for dogs and >150 ng/mL for cats; 12 dogs and 7 cats).

Urine cystatin B concentrations ranged from 9 to 2128 ng/mL in dogs and from 0 to 154 ng/mL in cats. Using urine cystatin B concentrations, pets were classified as low (≤30 ng/mL for dogs and ≤20 ng/mL for cats; 14 dogs and 22 cats); elevated (31–350 ng/mL for dogs and 21–150 ng/mL for cats; 9 dogs and 7 cats), and high (>350 ng/mL for dogs and >150 ng/mL for cats; four dogs and one cat).

Twelve dogs (7 males and 5 females) and 14 cats (9 males and 5 females) had at least one high tissue damage biomarker. Urine clusterin and cystatin B concentrations were correlated (overall: r = +0.48; *P* = 0.0002; dog: r = +0.11; *P* = 0.59; cat: r = +0.39; *P* = 0.05). Six dogs (4 males and 2 females) and two cats (2 males) had high serum BAIB and urine clusterin concentrations. All four dogs with high urine cystatin B concentrations and three of the four dogs with high serum BAIB concentrations also had high urine clusterin concentrations.

#### Species and sex differences

Urine cystatin B and clusterin concentrations were higher in dogs compared with cats (both *P* < 0.0001), whereas serum BAIB concentrations were higher in cats (*P* = 0.03; **Fig 2**). Serum BAIB concentrations were higher in males than in females (*P* = 0.01; **Table 6**). Ten males vs. two females had high BAIB concentrations (*P* = 0.05).

**Table 6:** Serum and urine kidney tissue damage biomarkers before and after general anesthesia and dental cleaning procedures in cats and dogs with periodontal disease stratified by sex (n = 62)*.

Biomarkers of	Dental cleaning procedures			*P*-values^‡^	
Kidney Damage	1 week before	6 hours after	1 week after	6 hours after	1 week after
Serum (μg/dL)					
BAIB^§^					
Female	0 (0, 1.8)*	0 (0, 2.0)	0 (0, 2.4)	0.88^†^	0.92^†^
Male	2.0 (0, 6.5)	0 (0, 2.3)	1.0 (0, 2.9)	0.02	0.08
Urine (ng/mL)^§^					
Cystatin B					
Female	20 (10, 30)	22 (9, 45.5)	19.5 (7, 36)	0.45	0.34
Male	14 (8, 37)	21 (9.5, 44.5)	17 (10.5, 66.5)	0.25	0.56
Clusterin					
Female	142 (42, 300)	130 (35, 195)	84 (40, 255)	0.54	0.78
Male	120 (25, 391)	114 (26, 227)	74 (27, 211)	0.94	0.08

*Median (Q25, Q75). 14 male neutered dogs, 17 female spayed dogs; 9.5 ± 2.1 years (range: 3–14 years old. 19 male neutered cats, 12 female spayed cats; 9.7 ± 3.5 years (range: 2–16 years old).

^‡^A paired Wilcoxon rank sum test was used to determine *P* values for changes from 1 week before the procedure.

Male and female differences were calculated using Wilcoxon rank sum test, with 0.05 < *P* < 0.10 denoted by ^†^, 0.01 < *P* < 0.05 denoted by *, 0.001 < *P* < 0.01 denoted by **, and *P* < 0.001 denoted by ***.

^§^BAIB, ß-aminoisobutyric acid.

#### Age effects

Using Spearman correlations, urine clusterin (r = +0.39; *P* = 0.003) and cystatin B (r = +0.27; *P* = 0.04) concentrations increased with age. Animals of at least 9 years of age had higher urine clusterin (*P* = 0.01) and cystatin B (*P* = 0.03) values than younger animals (**Table 7**). High urine clusterin concentrations were observed in 13 of 20 ≥11-years-old animals vs. five of 32 younger animals (*P* = 0.0007).

**Table 7:** Serum and urine kidney tissue damage biomarkers before and after general anesthesia and dental cleaning procedures in cats and dogs with periodontal disease stratified by age (n = 62)*.

Biomarkers of	Dental cleaning procedures			*P*-values^‡^	
Kidney Damage	1 week before	6 hours after	1 week after	6 hours after	1 week after
Serum (μg/dL)					
BAIB^§^					
<9 years old	1.9 (0, 6.8)	0 (0, 2.05)	0 (0, 5.2)	0.16	0.35
≥9 years old	0.9 (0, 2.3)	0.35 (0, 2.3)	0.9 (0, 2.6)	0.26	0.33
Urine (ng/mL)^§^					
Cystatin B					
<9 years old	12 (5, 32.5)*	11 (8, 25)*	13 (8, 21)*	0.45	0.42
≥9 years old	22.5 (13, 67)	30 (11, 77)	24 (11, 65)	0.29	0.72
Clusterin					
<9 years old	73 (16, 161)*	80 (12, 172)	69 (26, 191)	0.33	0.54
≥9 years old	179 (39, 410)	142 (36, 306)	95 (37, 310)	0.80	0.13

*Median (Q25, Q75). 14 male neutered dogs, 17 female spayed dogs; 9.5 ± 2.1 years (range: 3–14 years old. 19 male neutered cats, 12 female spayed cats; 9.7 ± 3.5 years (range: 2–16 years old).

^‡^A paired Wilcoxon rank sum test was used to determine *P* values for changes from 1 week before the procedure.

Differences due to age of the animal were calculated using Wilcoxon rank sum test, with 0.05 < *P* < 0.10 denoted by ^†^, 0.01 < *P* < 0.05 denoted by *, 0.001 < *P* < 0.01 denoted by **, and *P* < 0.001 denoted by ***.

^§^BAIB, ß-aminoisobutyric acid.

#### Kidney tissue damage and kidney function biomarkers are linked

High urine cystatin B was correlated with high BUN (dogs: r = +0.52; *P* = 0.006 and cats: r = +0.55; *P* = 0.002) and elevated UPC ratios (dogs: r = +0.59; *P* = 0.001 and cats: r = +0.63; *P* = 0.0002). Urine cystatin B concentrations (*P* = 0.02) were higher in animals with at least one vs. no abnormal kidney function biomarkers (**Table 8**). High urine cystatin B concentrations were only observed in animals with at least one abnormal kidney function biomarker (*P* = 0.02). Most animals with normal kidney function biomarker concentrations had low urine cystatin B concentrations (21 of 25), whereas only 14 of 31 animals with abnormal kidney function biomarker concentrations had low urine cystatin B concentrations (*P* = 0.005). Similarly, most animals with normal kidney function biomarker concentrations had low urine clusterin concentrations (16 of 25), whereas only 8 of 31 animals with abnormal kidney function biomarker concentrations had low urine clusterin concentrations (*P* = 0.006).

**Table 8:** Serum and urine kidney tissue damage biomarkers before and after general anesthesia and dental cleaning procedures in cats and dogs with periodontal disease stratified by abnormal kidney function biomarkers prior to procedure (n = 62)*.

Biomarkers of	Dental cleaning procedures			*P*-values^‡^	
Kidney Damage	1 week before	6 hours after	1 week after	6 hours after	1 week after
Serum (μg/dL)					
BAIB^§^					
Normal Kid.	0.9 (0, 4.0)	0 (0, 2.1)	0.4 (0, 2.0)	0.13	0.09
Abnormal Kid.	1.6 (0, 2.1)	0 (0, 2.4)	0.7 (0, 2.9)	0.35	0.75
Urine (ng/mL)^§^					
Cystatin B					
Normal Kid.	14 (8, 19)*	16 (9, 30)^†^	15 (9, 44)	0.17	0.28
Abnormal Kid.	30 (13, 52)	28 (11, 77)	21 (12, 37)	0.51	0.31
Clusterin					
Normal Kid.	42 (23, 220)	49 (14, 219)^†^	37 (15, 115)*	0.76	0.42
Abnormal Kid.	168 (75, 352)	151 (52, 216)	158 (55, 252)	0.83	0.64

*Median (Q25, Q75). 14 male neutered dogs, 17 female spayed dogs; 9.5 ± 2.1 years (range: 3–14 years old. 19 male neutered cats, 12 female spayed cats; 9.7 ± 3.5 years (range: 2–16 years old).

^‡^A paired Wilcoxon rank sum test was used to determine *P* values for changes from 1 week before the procedure.

Differences due to abnormal kidney function biomarkers were calculated using Wilcoxon rank sum test, with 0.05 < *P* < 0.10 denoted by ^†^, 0.01 < *P* < 0.05 denoted by *, 0.001 < *P* < 0.01 denoted by **, and *P* < 0.001 denoted by ***.

^§^Kid., kidney function biomarker; BAIB, ß-aminoisobutyric acid.

#### Kidney tissue damage biomarkers are linked with severity of dental disease

Severity of dental disease one week prior to dental procedures (based on subsequent procedures performed) and elevated urine cystatin B and clusterin values were linked (**Table 9**). Length of anesthesia was correlated with higher urine cystatin B concentrations (overall: r = +0.35; *P* = 0.008) and clusterin concentrations (overall: r = +0.34; *P* = 0.01). Higher urine concentrations of cystatin B (overall: *P* = 0.003) and clusterin concentrations (overall: *P* = 0.0005) were observed in animals with vs. without ≥60 min anesthesia one week later. High urine cystatin B concentrations (n = 5) were observed exclusively in dogs (n = 21) and cats (n = 16) with ≥60 min anesthesia time. Except for two cats, high urine clusterin concentrations (n = 19) were observed exclusively in dogs and cats with ≥60 min anesthesia (*P* = 0.002). We observed higher urine concentrations of cystatin B (*P* = 0.04), but not urine clusterin concentrations (dogs: *P* = 0.13) in dogs with vs. without tooth extraction.

**Table 9:** Serum and urine kidney tissue damage biomarkers before and after general anesthesia and dental cleaning procedures in cats and dogs with periodontal disease stratified by length of anesthesia (n = 62)*.

Biomarkers of	Dental cleaning procedures			*P*-values^‡^	
Kidney Damage	1 week before	6 hours after	1 week after	6 hours after	1 week after
Serum (μg/dL)					
BAIB^§^					
<60 min Anesth.	1.8 (0, 6.8)	0 (0, 2.5)	1.2 (0, 3.5)	0.11	0.36
≥60 min Anesth.	0.7 (0, 2.2)	0 (0, 2.1)	0 (0, 2.3)	0.36	0.35
Urine (ng/mL)^§^					
Cystatin B					
<60 min Anesth.	10.5 (5.5, 27)**	11 (8.5, 25.5)**	12 (8.5, 39.5)	0.20	0.98
≥60 min Anesth.	25 (14, 52)	27 (11.5, 51.5)	21.5 (12, 42)	0.38	0.81
Clusterin					
<60 min Anesth.	37 (8, 139)***	33 (14, 133)***	39 (18, 140)**	0.91	0.80
≥60 min Anesth.	205 (75, 412)	166 (76, 320)	136 (54, 313)	0.83	0.25

*Median (Q25, Q75). 14 male neutered dogs, 17 female spayed dogs; 9.5 ± 2.1 years (range: 3–14 years old. 19 male neutered cats, 12 female spayed cats; 9.7 ± 3.5 years (range: 2–16 years old).

^‡^A paired Wilcoxon rank sum test was used to determine *P* values for changes from 1 week before the procedure.

Differences due to anesthesia length were calculated using Wilcoxon rank sum test, with 0.05 < *P* < 0.10 denoted by ^†^, 0.01 < *P* < 0.05 denoted by *, 0.001 < *P* < 0.01 denoted by **, and *P* < 0.001 denoted by ***.

^§^Anesth., anesthesia; BAIB, ß-aminoisobutyric acid.

### Kidney biomarker concentrations in dogs and cats six hours after dental procedures

#### Kidney function biomarkers

The number of dogs with at least one abnormal kidney function biomarker remained at 18, whereas the number of cats with at least one abnormal kidney function biomarker increased from 16 before the procedure to 24 thereafter (*P* = 0.06). Compared with one week before dental procedures, dogs had lower BUN concentrations shortly after dental procedures (22 of 30 dogs had lower values; median: -5 mg/dL; *P* < 0.0001; **Fig 1; Table 1**). The same was true for cats (20 of 30 cats had lower values; median: -2 mg/dL; *P* = 0.007).

#### Species differences

The responses for the other four kidney function biomarkers differed between dogs and cats. Serum SDMA concentrations increased more often in cats (29 of 30 cats had higher values; median: +4 μg/dL; *P* < 0.0001) compared with dogs (18 of 30 dogs had higher values; median: +2 μg/dL; *P* = 0.08). The number of cats with abnormal SDMA concentration increased from 4 to 15 (*P* = 0.005) and in dogs from 5 to 6. Serum Cr concentrations decreased after the procedure in dogs (22 of 30 dogs had lower values; median: -0.15 μg/dL; *P* < 0.0001), but not in cats (10 of 30 cats had lower values; +0.1 μg/dL; *P* = 0.15).

The USG values decreased after dental procedures in cats (21 of 30 cats had lower values; median: -0.007; *P* = 0.002), but not in dogs (15 of 30 dogs had lower values; median: -0.0005; *P* = 0.44). The UPC ratio increased in five dogs and decreased in one dog; whereas the UPC ratio increased in four cats and decreased in eight cats (*P* = 0.05 for species differences).

#### Sex and age differences

Serum Cr decreased in females (*P* = 0.04) but not in males (*P* = 0.66), whereas USG values decreased in males (*P* = 0.01) but not in females (*P* = 0.25; **Table 2**). Serum Cr decreased in ≥9-yr old animals (*P* = 0.003) but not in younger animals (*P* = 0.37); in contrast, UPC ratios tended to increase in ≥9-yr old animals (*P* = 0.09) but not in younger animals (*P* = 0.29; **Table 3**).

#### Changes in kidney function biomarker concentrations are linked with UPC ratios one week before the procedure

Declines in kidney function biomarker concentrations centered around changes in SDMA concentrations. Increases in SDMA concentrations were linked to both decreased USG values (overall: r = -0.34; *P* = 0.01) and increased Cr concentrations (overall: r = +0.33; *P* = 0.01). Increases in SDMA concentrations were linked to elevated UPC ratios 1 week before the procedure (overall: r = +0.28; *P* = 0.03). Animals with elevated UPC ratios 1 week before the procedure had greater increases in SDMA concentrations compared with those without (*P* = 0.02). Decreases in Cr concentrations were linked to decreases in BUN concentrations (overall: r = +0.28; *P* = 0.03) and increases in UPC values (overall: r = -0.27; *P* = 0.04).

#### Changes in kidney function biomarker concentrations are linked with anesthesia duration

A decline in Cr concentrations was observed in cats and dogs with ≥60 min anesthesia times (*P* = 0.04), but not in cats and dogs with shorter anesthesia times (*P* = 0.73; **Table 4**). The decline in BUN concentrations was larger in dogs and cats with ≥60 min vs. shorter anesthesia times (*P* < 0.0001 vs. *P* = 0.004). In contrast, UPC value differences in dogs and cats with ≥60 min vs. shorter anesthesia times were larger 6 hours after vs. 1 week before the procedure (increased from *P* = 0.01 to *P* = 0.0004), as more dogs and cats with ≥60 min anesthesia times had elevated UPC values.

The impact of anesthesia duration on SDMA and Cr changes differed between cats and dogs. Cats with ≥60 min anesthesia times had greater SDMA increases compared with cats with shorter anesthesia times (*P* = 0.02), whereas duration of anesthesia did not impact SDMA changes in dogs (*P* = 0.60). Dogs with ≥60 min anesthesia times tended to have greater Cr declines compared with the other dogs (*P* = 0.09), whereas duration of anesthesia did not impact Cr changes in cats (*P* = 0.98). Moreover, anesthesia time was correlated with Cr changes in dogs (r = -0.50; *P* = 0.005), but not in cats (r = +0.00; *P* = 0.98).

#### Kidney tissue damage biomarkers

The number of dogs with at least one high tissue damage biomarker decreased from 12 to 8 dogs (no new dogs with high tissue damage biomarkers) and the number of cats with at least one high tissue damage biomarker decreased from 14 to 12. After the dental cleaning procedures, the only kidney tissue damage biomarker significantly changed was serum BAIB (*P* = 0.03) in dogs but not in cats (*P* = 0.57; **Fig 2**). Serum BAIB decreased in 10 dogs (4 females and 6 males) and increased in five dogs (4 males and one female). The number of dogs with elevated and high concentrations of BAIB decreased from 10 to 6 and from 4 to 1, respectively.

#### Sex and age differences

Serum BAIB concentrations decreased in male animals (*P* = 0.02; 7 dogs and 9 cats decreased) but not in female animals (*P* = 0.88; 4 dogs and 3 cats decreased; **Table 6**), which was linked to the fact that more male than female (22 vs. 12) animals had detectable BAIB concentrations before the procedure. We did not observe age-related differences for changes in kidney tissue damage biomarkers (**Table 7**).

#### Changes in kidney tissue damage and kidney function biomarkers are linked

Increases in urine clusterin concentrations were linked with increases in USG values (overall: r = +0.39; *P* = 0.003), and increases in urine cystatin B concentrations were linked with increases in BUN concentrations (overall: r = +0.36; *P* = 0.009).

Increases in urine cystatin B concentrations were linked with higher concentrations of SDMA (overall: r = +0.34; *P* = 0.01) and Cr (overall: r = +0.35; *P* = 0.008) 1 week before the procedure. Increases in urine clusterin concentrations tended to be linked to higher concentrations of SDMA (r = +0.34; *P* = 0.09) and Cr (r = +0.35; *P* = 0.08) 1 week before the procedure in dogs, but lower concentrations of SDMA (r = -0.31; *P* = 0.10) and Cr (r = -0.21; *P* = 0.28) 1 week before the procedure in cats.

#### Changes in kidney tissue damage biomarkers are linked with severity of dental disease in cats

There was a general decrease in kidney tissue damage biomarker concentrations with shorter anesthesia times in cats (BAIB: r = +0.28; *P* = 0.14; urine cystatin B: r = +0.30; *P* = 0.11; urine clusterin: r = +0.34; *P* = 0.08). Cats with anesthesia time ≥60 min differed (*P* = 0.03) in their urine clusterin response from cats with anesthesia time <60 min; the former generally experiencing increases whereas the latter experienced decreases in urine clusterin concentrations. Similarly, urine clusterin concentrations increased in cats with tooth extraction(s) (median = +24; *P* = 0.02), and decreased in cats with dental cleaning only (median = -9; *P* = 0.05). The number of cats with elevated or high urine cystatin B concentration increased from 5 to 10 in cats with ≥60 min anesthesia and decreased from 3 to 1 in cats with shorter anesthesia times. The number of cats with high BAIB concentrations increased from 3 to 5 cats with ≥60 min anesthesia and decreased from 5 to 3 cats with shorter anesthesia times.

### Kidney biomarker concentrations in dogs and cats one week after dental procedures

#### Kidney function biomarkers

The number of dogs with at least one abnormal kidney function biomarker decreased from 18 before the dental procedure to 13 after the procedure, whereas the number of cats with at least one abnormal kidney function biomarker increased from 16 to 18. The only biomarker that changed significantly compared with one week before dental procedures was SDMA in cats (17 of 31 cats had higher concentrations; median: +1 μg/dL; *P* = 0.02; **Fig 1; Table 1**). This change was also observed 6 hours after the dental procedure. The number of cats with abnormal SDMA concentrations increased from 4 to 8 (4 new). The four original cats with abnormal SDMA concentrations that persisted had at least two abnormal kidney function biomarkers before the dental procedure. The four new cats with abnormal SDMA concentrations (2 males and 2 females) 1 week after the procedure had already abnormal SDMA concentrations 6 hours after the dental procedure. The number of dogs with abnormal SDMA concentrations decreased from 6 to 4 (4 less and 2 new). The two original dogs that had persistently increased SDMA concentrations had at least two abnormal kidney function biomarkers throughout the study.

Increases in SDMA concentration were linked with elevated UPC ratios 1 week before dental cleaning (overall: r = +0.22; *P* = 0.09). The two new dogs that developed abnormal SDMA concentrations after the procedure had persistent proteinuria throughout the study, and all dogs with an SDMA increase of at least 0.4 μg/dL had at least borderline proteinuria. Three of the four cats that developed elevated SDMA concentrations had borderline proteinuria, and three of the four cats with an SDMA increase of at least 0.4 μg/dL had at least borderline proteinuria.

All 16 cats and the two dogs with Cr concentrations ≥1.4 mg/dL before the procedure had serum Cr concentrations ≥1.3 mg/dL one week after the procedure. Five cats had an increase of ≥0.3 mg/dL in Cr between 1 week before and 1 week after the procedure; all but one had borderline proteinuria before the procedure.

The six animals (3 dogs and 3 cats) with proteinuria before the dental procedure remained proteinuric and the number of animals with borderline proteinuria did not change. No animal developed proteinuria after the procedure.

Four dogs with isosthenuria before the procedure remained isosthenuric (n = 2) or borderline isosthenuric (USG = 1.021). One dog with borderline proteinuria before the procedure (UPC = 0.3) developed isosthenuria (USG = 1.011) after the procedure. Six of the nine cats with isosthenuria before the procedure remained isosthenuric. Two cats that developed borderline proteinuria after the procedure developed isothenuria.

#### Species differences

Concentrations of SDMA increased in cats (*P* = 0.02) but not in dogs (*P* = 0.97), as the number of cats with abnormally high SDMA concentrations increased from 4 to 8 cats and remained unchanged at one in dogs (**Fig 1; Table 1**). Species differences in USG values decreased in significance from *P* = 0.006 before the cleaning procedure to *P* = 0.04 after, as more cats had USG values above 1.060 one week after the procedure (**Fig 1**).

#### Sex and age differences

In general, improvements in kidney function biomarker values were primarily observed in <9 year-old animals, whereas older animals more often had no change or worse values (**Table 3**). Concentrations of BUN decreased in <9 year-old animals (median: -1 mg/dL; *P* = 0.05) but remained the same in older animals (*P* = 0.27). Similarly, UPC ratios decreased in <9 year-old animals (all animals with elevated UPC ratios had decreased values; *P* = 0.04) but remained the same in older animals (*P* = 0.86). Creatinine concentrations increased in ≥9 year-old animals (median: +0.1 mg/dL; *P* = 0.05) but remained the same in younger animals (*P* = 0.27). No sex differences were observed for change in kidney function biomarker values (**Table 2**).

#### Changes in kidney function biomarkers are linked with severity of dental disease

Declines in USG values were linked to length of anesthesia (**Fig 2; Table 4**). Animals with ≥60 min anesthesia length differed in their USG response (*P* = 0.05) from those with <60 min anesthesia length; the former generally experiencing decreases (median: -0.002) whereas the latter experienced increases in USG values (median: +0.002). Concentrations of SDMA increased from one week before to one week after dental cleaning procedures in cats with tooth extraction(s) (*P* = 0.004) and in cats with ≥60 min anesthesia time (*P* = 0.007), whereas no changes were observed in cats with dental cleaning only (*P* = 0.94) and in cats with shorter anesthesia time (*P* = 0.68).

#### Dental procedures are linked to both improvement and deterioration of kidney function biomarkers

We observed both improvement and deterioration of kidney function biomarkers values after the dental procedures. Abnormal kidney function biomarker values persisted in animals that had increased SDMA concentrations in combination with an elevated Cr concentration before the study (two dogs and four cats). Within this group, two dogs and two cats also had elevated BUN concentrations, one dog and three cats also had borderline proteinuria, and all four cats had isosthenuria.

Deterioration of kidney function was linked to elevated UPC ratios before the procedure and was reflected in SDMA concentrations. All six animals (2 dogs and 4 cats) that developed elevated SDMA concentrations after the procedure had either proteinuria of renal or post-renal origin at all three sampling times (two dogs and one cat; positive bacterial growth from urine cultures; WBC, epithelial cells, and occasional hyaline casts in the urine), proteinuria six hours after the procedure (one cat; UPC = 1.9), or borderline proteinuria (two cats; UPC = 0.2) at all three sampling times.

Improvements in kidney function biomarker values were linked to those animals with fewer abnormal kidney function biomarkers. Eight dogs (4 males and 4 females) and three cats (1 male and 2 females) changed from abnormal to normal kidney function biomarker concentrations. Six dogs had normal UPC ratios throughout the study and two dogs had UPC ratios of 0.2, and one cat had normal UPC ratios throughout the study and two cats had UPC ratios of 0.2.

#### Kidney tissue damage biomarkers

There was a general trend of decreased kidney tissue damage biomarker concentrations after the procedure. The number of dogs with at least one high kidney tissue damage biomarker concentration decreased after the procedure from 12 to 6. Nine dogs changed from high to normal concentrations of kidney tissue damage biomarkers (6 males and 3 females), whereas three dogs (two males and one female) changed from normal to high concentrations of kidney tissue damage biomarkers. The number of dogs with high BAIB concentration decreased from four to one; the number of dogs with high cystatin B concentration decreased from four to one; and the number of dogs with high clusterin concentration decreased from 12 to six (**Fig 2; Table 5**). All six dogs with high kidney tissue damage biomarker concentrations had increased SDMA concentrations (range: +2 to +13 μg/dL), five dogs had USG values ≤1.027, and four dogs had decreased USG values and SDMA concentrations ≥14 μg/dL.

The number of cats with at least one high kidney tissue damage biomarker concentration decreased from 14 to 11. Four cats changed from high to normal kidney tissue damage biomarker concentrations (2 males and 2 females) and one female cat changed from normal to high concentrations of kidney tissue damage biomarkers. The number of cats with high BAIB concentration decreased from eight to six; the number of cats with high cystatin B concentration decreased from one to zero; and the number of cats with high clusterin concentration decreased from seven to six. In contrast to dogs, there were limited common features among cats with high kidney tissue damage biomarkers.

#### Sex and age differences

We tended to observe decreased BAIB concentrations in males (*P* = 0.08; 7 dogs and 10 cats had decreased BAIB concentrations vs. 2 dogs and 5 cats had increased BAIB concentrations) but not in females (*P* = 0.92; 3 dogs and 3 cats had decreased BAIB concentrations vs. 6 dogs and 5 cats had increased BAIB concentrations; **Table 6**). We also tended to observe decreased urine clusterin concentrations in males (*P* = 0.08; 8 dogs and 12 cats had decreased urine clusterin values vs. 4 dogs and 7 cats had increased urine clusterin values) but not in females (*P* = 0.78; 6 dogs and 3 cats had decreased urine clusterin values vs. 8 dogs and 7 cats had increased urine clusterin values). No age differences in kidney tissue damage biomarker responses were observed (**Table 7**).

#### Changes in kidney tissue damage and kidney function biomarkers are linked

Decreases in urine clusterin values were linked to increases in BUN concentrations (r = -0.32; *P* = 0.02). Decreases in BAIB concentrations were linked to increases in USG values (r = -0.27; *P* = 0.05). Decreases in urine cystatin B values were linked to elevated UPC ratios before the dental procedure (r = -0.32; *P* = 0.02).

We observed decreased serum BAIB concentrations in animals with normal kidney function biomarkers (*P* = 0.09; 4 dogs and 7 cats had decreased vs. 2 dogs and 4 cats had increased BAIB concentrations), but not in animals with abnormal kidney function biomarkers before the study (*P* = 0.75; 6 dogs and 5 cats had decreased vs. 6 dogs and 6 cats had increased BAIB concentrations; **Fig 2; Table 8**). Differences in urine cystatin B concentrations between animals with vs. without abnormal kidney function biomarkers before the study decreased from *P* = 0.02 to *P* = 0.42, as less animals with elevated kidney function biomarkers before the study had high urine cystatin B concentrations one week after the procedure (the number of animals with high urine cystatin B concentrations decreased from 5 to 1). In contrast, differences in urine clusterin concentrations between animals with vs. without abnormal kidney function biomarkers before the study increased from *P* = 0.12 to *P* = 0.01, as less animals with normal kidney function biomarkers before the study had high urine clusterin concentrations after the procedure (the number of dogs with high urine clusterin concentrations decreased from 6 to 0).

#### Changes in kidney tissue damage biomarkers are linked with severity of dental disease

Differences in urine cystatin B concentrations between animals with vs. without ≥60 min anesthesia times decreased from *P* = 0.003 before the procedure to *P* = 0.30 one week after the procedure, and between animals with vs. without tooth extraction from *P* = 0.07 to *P* = 0.46.

## Discussion

Dental disease, both prevalence and severity, are established risk factors for CKD [15–17]. Routine veterinary visits for management of periodontal disease provide an opportunity to assess renal function and detect early stages of CKD in cats and dogs that appear physically healthy. Current knowledge about the prevalence of abnormal kidney function biomarkers indicative of early stages of CKD in asymptomatic dogs and cats with periodontal disease is limited, and was the first objective of this study.

A biomarker is a functional or biochemical indicator of a disease process that has diagnostic and/or prognostic utility [7]. Ideally, samples can be easily obtained and measured accurately and reproducibly [7]. For the purpose of this study, renal biomarkers were analyzed using serum and urine samples. Renal function biomarkers for CKD included serum SDMA, Cr, and BUN concentrations, and urine UPC ratio and USG. Biomarkers for renal tissue damage included serum BAIB concentrations, and urine cystatin B and kidney-specific clusterin concentrations.

### Prevalence of CKD in asymptomatic dogs and cats referred for dental cleaning procedures

In the present study, a total of 18 dogs (58%) had at least one abnormally high kidney function biomarker and three dogs had at least two abnormally high kidney function biomarkers. Two dogs were classified IRIS stage 2, eight dogs were classified IRIS stage 1 (half for elevated SDMA concentrations and half for isosthenuria), three dogs had proteinuria, and six dogs had borderline proteinuria. A total of 16 cats (52%) had at least one abnormally high kidney function biomarker and three cats had at least two abnormally high kidney function biomarkers. One cat was classified IRIS stage 3 (Cr: 2.9 mg/dL, SDMA: 16 μg/dL, BUN: 35 mg/dL; borderline proteinuria, and isosthenuria), ten cats were classified IRIS stage 2, three cats were classified IRIS stage 1 (all for isosthenuria), three cats had proteinuria, and ten cats had borderline proteinuria. In the current study, pets were selected based on a healthy physical exam. With more advanced stages of CKD (Stage 3 and Stage 4), nearly all cats and dogs are symptomatic, as ≥75% of kidney function is lost [18]. Thus, having only one cat and no dogs with IRIS stage 3 CKD was within expectations for this study.

Pets with IRIS stage 2 CKD consisted of those with elevated SDMA and Cr concentrations (2 dogs and 3 cats) and those with only elevated Cr concentrations (5 cats). Serum Cr has limited value as a biomarker of CKD in early stages of CKD because it does not increase above the reference interval until approximately 60% of nephrons in cats [19] and 75% of nephrons in dogs [20] are nonfunctioning. Based on normal references intervals established in healthy cats, IDEXX uses a higher cut-off point for abnormally high Cr concentrations in cats of ≥2.4 mg/dL instead of ≥1.6 mg/dL as stated in IRIS guidelines [14]. Increasing Cr concentrations over time indicates declining kidney function and improves diagnostic accuracy of Cr in early stages of CKD. We observed six cats and no dogs with ≥0.3 mg/dL serum Cr increase over the 2-week study, including the IRIS stage 3 cat and two cats that developed elevated SDMA concentrations during the study. The current study supports a grouping of cats with both elevated SDMA and elevated Cr concentrations (according to IRIS guidelines [14]) as IRIS stage 2 CKD. These four cats also had isosthenuria, and three of four cats had borderline proteinuria, indicative of a more advanced stage of kidney dysfunction.

Pets with IRIS stage 1 CKD consisted of those with elevated SDMA concentration and those with isosthenuria (≤1.020 in dogs and ≤1.030 in cats). The value of USG as an early stage CKD biomarker is limited, because over-hydration and several non-renal diseases can also result in isosthenuria. Moreover, isosthenuria is detectable once approximately 67% of nephrons are not functioning. In comparison, the upper limit of the normal reference interval for SDMA of <14 μg/dL corresponds to a decrease in GFR of 24% of that for healthy cats [19], and a decrease in GFR of 49% from mean GFR for healthy dogs [20]. Thus, elevated SDMA concentrations can help identify earlier stages of CKD than either Cr or USG values. In the present study, six dogs and four cats had elevated SDMA concentrations (four dogs had only elevated SDMA concentrations) indicative of IRIS stage 1. Marked increases in SDMA concentration over time indicate declining kidney function and improves the diagnostic accuracy of SDMA in early stage CKD. We observed eight cats and six dogs with ≥3 μg/dL increases in SDMA over the 2-week study, with all increasing to at least 13 μg/dL.

The IRIS guidelines use UPC ratios to substage CKD based on proteinuria [14]. The UPC ratio reflects the amount of protein lost in the urine relative to hydration status. Having a UPC ratio greater than 0.5 in dogs (0.4 in cats) is consistent with proteinuria, and a UPC ratio between 0.2–0.5 in dogs (0.2–0.4 in cats) is considered borderline proteinuria [14]. Elevated UPC ratios indicate acute inflammation and tissue damage that could have a pre-renal, renal (i.e., primary glomerular disease), or infectious or hemorrhagic post-renal cause making UPC a nonspecific biomarker for an active inflammatory process. Thus, elevated UPC ratios could provide a link between dental diseases and abnormal renal function. In the present study, three cats and three dogs had proteinuria, but none had elevated SDMA concentrations. Geriatric dogs and cats with proteinuria are at increased risk for developing CKD [21]. Thus, cats and dogs with proteinuria but normal SDMA concentrations may be at increased risk for developing CKD because of progressive renal tissue damage. Six dogs and ten cats had borderline proteinuria, including one IRIS stage 3 cat, two IRIS stage 2 cats and 2 IRIS stage 2 dogs. Elevated UPC ratios have been linked to progressive vs. stable CKD [22]. Thus, cats and dogs with elevated SDMA and UPC ratios may be at increased risk for CKD progression, and the combination may distinguish between stable CKD (only SDMA increased) and progressive CKD (both increased).

Because pets with CKD can remain asymptomatic until advanced stages, detection of CKD using biomarkers is essential for early diagnosis and therapeutic interventions. Our findings are consistent with what has been reported previously for the general dog and cat populations [1, 2] and should alert practitioners to check for increasing and/or persistent elevations in kidney function biomarkers in aging dental patients. It is recommended that IRIS staging of CKD be based on assessments on at least two occasions at least two weeks apart in hydrated, stable patients [14].

### Relevance of increased renal tissue damage biomarker concentrations in asymptomatic cats and dogs

Given that the UPC ratio is a nonspecific inflammation biomarker, development of kidney-specific tissue damage biomarkers are at the forefront of veterinary medicine [7]. We evaluated three novel biomarkers, serum BAIB and urine cystatin B and kidney-specific clusterin, and their prevalence in asymptomatic dogs and cats referred for dental procedures.

Serum BAIB is a “hybrid” kidney function and tissue damage biomarker. ß-aminobutyric acid (BAIB) is an amine catabolite resulting from either thymine or valine degradation, and is usually present below the detection limit in blood [7]. In the current study, 16 dogs (52%) and 10 cats (32%) had non-detectable serum BAIB concentrations before the dental procedures. Serum BAIB can increase because of inadequate glomerular filtration or inflammation in combination with impaired renal tubular catabolism [23, 24]. Inadequate glomerular filtration links serum BAIB to serum Cr, BUN, and SDMA. Injury to tubular epithelial cells by nephrotoxins results in BAIB accumulation in blood [7], indicating that serum BAIB is an indicator of tubular dysfunction. Recently, we reported that in healthy cats serum BAIB concentrations were at or below the detection limit, whereas cats with CKD had elevated BAIB concentrations [25]. Limitations of serum BAIB as a kidney tissue damage biomarker are that poor glomerular filtration can result in high serum BAIB concentrations and that poor tubular resorption can result in low serum BAIB concentrations. In this study, BAIB concentrations stratified pets into three groups: non-detectable, low (0.1–4 μg/dL), and high (4.1–31 μg/dL). We observed four dogs and eight cats with BAIB concentrations >4 μg/dL.

In comparison to serum BAIB, elevated urine clusterin and cystatin B concentrations indicate an active inflammatory process (clusterin) and tissue damage (cystatin B) in the kidneys. Not surprisingly, urine clusterin and cystatin B were correlated in the present study. The kidney-specific isoform of clusterin (also known as apolipoprotein J) is a multifunctional, ubiquitous, highly glycosylated extracellular protein that promotes renal tissue repair after renal tissue damage by down-regulating fibrosis [7]. Thus, urine clusterin is more of a kidney-specific acute phase protein biomarker, which indicates an active inflammatory process in the kidney. Cystatin B is a ubiquitous, small (11.1 kD), monomeric, non-glycosylated nuclear cysteine proteinase inhibitor [7]. In comparison with the better known kidney biomarker cystatin C, cystatin B is smaller and is only released in the blood stream when proximal tubular cells are damaged [7]. Thus, urine cystatin B is a more kidney-specific tissue damage biomarker, and indicates acute proximal tubular tissue damage. In support, urine clusterin and cystatin B concentrations are elevated for 5 days after nephrotoxin exposure in dogs and remain elevated for at least another week [7, 26]. Furthermore, both increase several days before serum Cr concentrations increase, indicating that urine clusterin and cystatin B are earlier indicators of nephrotoxicity, as well as indicators of milder forms of nephrotoxicity than are elevated serum Cr concentrations [7, 26].

Most urine biomarkers are normalized for urine dilution status using urine Cr concentrations in the denominator. However, in a previous study [27] it was shown that urine clusterin and cystatin B have nearly 6-fold lower concentrations than urine Cr, that both have right-skewed distributions vs. normal distributions for Cr, and that both can be below the detection limit. In addition, urine Cr concentration can also be altered in CKD. Thus, in our study we did not normalize urine clusterin and cystatin B concentrations in the urine.

In contrast to serum BAIB, 97% (344 out of 354) of the urine clusterin and cystatin B values in our study were above the detection level (the exceptions were one dog and one cat for cystatin B, and one dog and seven cats for clusterin). In comparison, others have reported that urine cystatin C was barely detectable in the urine of most healthy cats and dogs, although present in most cats and dogs with renal disease [28, 29]. Thus, the advantages of measuring urine cystatin B (and clusterin) are that concentrations can be monitored in the normal low range in kidneys lacking active inflammation and tissue damage, and then concentrations should be high with active renal inflammation and tissue damage, irrespective of presence or stage of CKD.

Compared with serum BAIB, urine clusterin and cystatin B both had larger dynamic ranges. Urine clusterin was usually low but extended up to 2,313 ng/mL; urine cystatin B extended up to 2,128 ng/mL in cats and dogs. These are concentrations similar to those observed after nephrotoxin exposure in dogs [7, 26]. Urine cystatin B and clusterin concentrations stratified into three groups: low, elevated, and high concentrations. We observed 12 dogs with >350 ng/mL clusterin concentrations and 7 cats with >150 ng/mL clusterin concentrations. In addition, we observed four dogs with >350 ng/mL cystatin B concentrations and one cat with >150 ng/mL cystatin B concentrations. Those cut-offs are based on our results and may differ among other canine and feline populations.

Importantly, low urine cystatin B and clusterin concentrations were more common in pets with normal kidney function biomarker concentrations. High urine cystatin B and clusterin concentrations were more common in animals with abnormal kidney function biomarker concentrations. Furthermore, urine cystatin B concentrations were linked to elevated UPC ratios and BUN concentrations, suggesting that the UPC ratio is a nonspecific biomarker of tissue damage. It was previously shown that urine clusterin and cystatin B concentrations are elevated in dogs with IRIS stage 2 and 3 CKD compared with healthy dogs, with clusterin showing a gradual increase with advanced stages [30]. Thus, high urine cystatin B and clusterin concentrations may be linked to CKD.

In total, twelve dogs (39%) and 14 cats (45%) had at least one elevated kidney tissue damage biomarker, and six dogs and three cats had at least two elevated kidney tissue damage biomarkers, suggesting that kidney tissue damage is not uncommon in asymptomatic cats and dogs referred for dental procedures. Potentially, urine cystatin B and clusterin may be used to diagnose active kidney disease allowing for more appropriate therapeutic interventions and ultimately better supportive care.

### Risk factors for increased concentrations of renal biomarkers in asymptomatic cats and dogs

Several studies have evaluated risk factors for developing CKD in cats and dogs [15–17]. In our study, serum Cr and BUN concentrations were higher in cats than dogs. In addition, USG values were higher in cats than dogs, which may be related to lower water consumption in cats vs. dogs [31, 32]. Less is known about risk factors for developing elevated serum BAIB and urine cystatin B and clusterin concentrations in dogs and cats. In the present study, urine cystatin B and clusterin values were considerably higher in dogs than cats, which is why we used separate cut-off values. In comparison to urine clusterin and cystatin B, differences were smaller in serum BAIB concentrations with cats having higher serum BAIB concentrations than dogs. Larger studies are needed to evaluate whether normal reference intervals may differ between cats and dogs.

In the present study, isosthenuria and borderline proteinuria were more common in females, and proteinuria, high Cr, SDMA, BUN, or BAIB concentrations were more common in male cats and dogs. Isosthenuria relates primarily to impairments in tubular reabsorption of water, whereas high Cr, SDMA, or BUN relate to impaired glomerular filtration. Serum BAIB is linked to Cr, SDMA, and BUN, and all must be excreted to avoid accumulation in the body.

In this cross-sectional study, we observed abnormally high SDMA, Cr, or BUN and urine cystatin B and clusterin concentrations primarily in older animals, which is consistent with results of larger surveys for kidney function biomarkers [6, 16]. Cobrin et al. [10] concluded that one of the limitations of serum cystatin C in dogs is its inconsistent link with age. The current study suggests that urine cystatin B and clusterin respond similar to SDMA, Cr, or BUN with age-dependent increases in concentrations. In cats, we also observed that advanced age was linked to lower USG, another indicator that older cats are at increased risk of developing CKD.

We were specifically interested in the link between kidney function biomarkers and dental disease severity. We observed the strongest link with UPC ratios, as elevated UPC ratios were associated with extended duration dental procedures, indicating a potential link between periodontal disease and compromised kidney function. This link was supported by the fact that high urine cystatin B and clusterin concentrations were also linked to extended duration dental procedures. Our results support the hypothesis that severe dental disease may increase the risk of renal tissue inflammation and damage. Thus, regular dental visits are necessary in older cats and dogs to manage dental diseases and prevent potential CKD.

### Immediate impact of dental cleaning procedures on renal function and tissue damage

Our second hypothesis was that dental cleaning procedures (i.e., dental scaling and polishing to remove the plaque and tartar from all tooth surfaces ± tooth extractions) performed under general anesthesia for extended periods (i.e., over 60 minutes) may result in renal tissue damage and decreased renal function within hours of the procedure. Dental procedures performed under general anesthesia have inherent risks including decreased cardiac output, leading to hypotension and decreased GFR [33]. Other risks associated with anesthesia include exposure to nephrotoxic drugs and acid-base imbalances in an animal that is already suffering from CKD [34]. The only consistent biomarker change across both species was a decrease in BUN six hours after dental procedures. In cats, serum SDMA increased and USG values decreased, whereas serum Cr and BAIB concentrations decreased in dogs.

There was a general trend of greater benefit for dogs with the cleaning procedures and greater risk for cats. Concerning is the increased number of cats with at least one abnormal kidney function biomarker (from 16 to 24) and the increased number with high SDMA concentrations (from 4 to 15) six hours after the procedure, as it raises the question of whether CKD is linked to the dental cleaning procedure in cats, specifically in high-CKD risk cats. Pets with elevated UPC ratios one week before the procedure, and cats with extended anesthesia times had greater increases in SDMA concentrations. Furthermore, there was a general decrease in kidney tissue damage biomarker concentrations in cats with shorter anesthesia times, whereas extended anesthesia times and tooth extractions increased kidney tissue damage biomarker concentrations, indicating a potential impact of dental disease on renal tissue damage. For example, bacteremia associated with the extraction procedure may damage the kidney.

Dogs had decreased serum Cr and BAIB concentrations immediately after the procedure. A potential explanation is that extra intravenous fluids promoted filtration of Cr and BAIB from the blood. In support, dogs with extended anesthesia times had greater serum Cr declines. The number of dogs with at least one high tissue damage biomarker decreased from 12 to 8 dogs. Older dogs were more likely to have decreased Cr concentrations, as they had higher Cr concentrations one week before. Serum BAIB concentrations decreased in males but not females, as male cats and dogs had higher BAIB concentrations compared with females one week before. However, the number of dogs with at least one abnormal kidney function biomarker remained the same at 18, as increases in UPC ratios were observed exclusively in older dogs with extended anesthesia times. In addition, five previously normal dogs developed abnormally high SDMA concentrations, isosthenuria, or both six hours after the procedure.

### Long-term impact of dental cleaning procedures on renal function and tissue damage

Given that each square centimeter of periodontal disease burden in dogs is linked to a 1.4 × greater likelihood of kidney pathology [11], our third hypothesis was that dental cleaning procedures performed under general anesthesia may result in improved renal function in dogs and cats long-term (i.e., one week after the cleaning procedures). In the present study, the general trend of greater benefits of dental cleaning procedures for dogs, but potential risks for cats observed 6 hours after the dental procedure remained one week after the cleaning procedures. The number of dogs with at least one abnormal kidney function biomarker decreased from 18 before the procedure to 13, and the number of dogs with at least one abnormal kidney tissue damage biomarker decreased from 12 to 6. In contrast, the number of cats with abnormal kidney function biomarker concentrations increased from 16 to 18, as the number of cats with abnormal SDMA concentrations increased from 4 to 8. Three of the 4 cats that developed elevated SDMA concentrations had borderline proteinuria one week before the cleaning procedures, which was the greatest risk factor for large increases in SDMA concentrations.

We also observed a general trend of greater benefit of dental procedures on kidney function biomarkers in low CKD-risk animals, yet a potential risk in high CKD-animals. Improvement in the concentrations of BUN and UPC ratios were observed in younger animals, whereas increases in serum Cr concentrations were observed in older animals.

Dental procedures may improve kidney function during early stages of CKD if there are no other complications. Eleven animals changed from abnormal to normal kidney function biomarker values with three having one week before UPC ratios of 0.2 and the remaining having one week before UPC ratios of 0.1. In contrast, animals with elevated one week before SDMA and Cr concentrations did not improve and had elevated concentrations at all three sampling times, indicating the importance of early intervention to prevent progression of CKD.

Progression of kidney disease occurs because of persistence of primary disease or addition of other renal insults [35]. In support, we observed eight cats and six dogs with ≥3 μg/dL SDMA increase and six cats and no dogs with ≥0.3 mg/dL Cr increase over the two week study, all had at least one elevated kidney tissue biomarker value, and all but three cats had elevated UPC ratios before or directly after the dental procedure. Early detection of injury and early medical intervention are critical to the outcome for these patients. In support, SDMA concentrations increased from one week before to one week after dental cleaning procedures in cats with tooth extraction(s) (*P* = 0.004), whereas no changes were observed in cats with dental cleaning only (*P* = 0.94), indicating the need for additional supportive care.

### Strengths and limitations of the study

Strengths of this study include the combination of traditional and novel serum and urine biomarkers to assess kidney function and tissue damage in cats and dogs referred for dental cleaning procedures. Novel serum biomarkers included BAIB, and novel urine biomarkers included cystatin B and kidney-specific clusterin. Regular dental visits provide an opportunity to detect asymptomatic bacterial urinary tract infections and CKD in dogs and cats. A combination of serum and urine biomarkers specific for kidney function and kidney tissue damage will give practitioners a more complete picture of kidney health before a procedure (e.g., general anesthesia and dental cleaning procedures) allowing for better patient management, better supportive care, and better clinical outcomes. Moreover, they can determine if an injury occurred during the procedure and provide additional supportive care after the procedure.

Limitations of this study included variations in age, breed, and sex of dogs and cats that are inherently associated with a prospective clinical study using client-owned pets. In addition, the severity of periodontal disease varied, necessitating tooth extraction(s) and longer anesthetic times in some pets. On the other hand, this allowed us the opportunity to document that tooth extraction(s) plus longer anesthesia times are a risk factor for kidney injury. The rate of intravenous fluid administration also varied, but in most cases was at or below the recommended maintenance fluid rate, thus avoiding the effects of hypervolemia on serum and urine biomarker concentrations. Blood pressure measurements were not consistently performed; thus, it was not possible to determine IRIS substaging by blood pressure. Another limitation was the short (one week) follow up period. It is possible, using renal biomarkers, that more benefits of dental cleaning would have been noted in a longer follow up period.

## Conclusions

In this study, we present evidence for a link between periodontal disease, biomarkers of renal tissue damage, and markers of compromised kidney function. Early stages of kidney disease are not uncommon in asymptomatic dogs and cats referred for dental cleaning procedures, specifically when they belong to a high-risk CKD group (older animals, male animals, animals with more severe dental disease). Dental cleaning procedures have the potential to improve kidney function in animals during early stages of CKD if there are no other complications. However, the disease will progress if the cause of kidney inflammation is not removed. Longer duration dental procedures in cats, specifically older animals, may carry inherent risks of kidney injury and impaired renal function. Future studies are warranted to confirm the results of our small study.

## Abbreviations used

ADMA: asymmetric dimethylarginine
BAIB: β-aminoisobutyric acid
BUN: blood urea nitrogen
CKD: chronic kidney disease
Cr: creatinine
GFR: glomerular filtration rate
IRIS: International Renal Interest Society
LCMS: liquid chromatography mass spectrometry
SDMA: symmetric dimethylarginine
UPC: urine protein: creatinine ratio
USG: urine specific gravity
